# Uterine Hemorrhage Avoided in Predisposed Women

**Published:** 1832-12

**Authors:** 


### UTERINE HEMORRHAGE.

### *Uterine Hemorrhage avoided in predisposed Women.*

Abridged from a Case communicated by Dr. KEY.

No events connected with medical practice occasion more anxiety to the practitioner, and alarm to the patient, than uterine hemorrhage; and, perhaps, none are more frequently the result of improper and injudicious interference. Uterine hemorrhage is often not to be avoided, and, with the most judicious management, frequently proves fatal; and the danger becomes awfully increased when delivery is intrusted to persons not fully conversant with the means of treating the extraordinary as well as the ordinary phenomena of labour; for the claims on the attendant's skill are in many cases so imperative, that there is no time for deliberation: he cannot avail himself of consultation, and has no reliance but in his own resources.

It is not, however, to such cases of hemorrhage that I am now anxious to engage attention; it is rather to those which supervene on delivery, and are often the consequence of an officious interference, or, as Dr. BLUNDELL has significantly called it, "a meddlesome midwifery." Many cases of hemorrhage arising from such practice I have seen, some of which were difficult to restrain, and others fatal. I am aware that it is bordering on a truism to observe, (and yet truths cannot be too frequently repeated,) that every structural arrangement for effecting parturition is, in well-formed women, admirably adapted to effect the purposed end: the uterus, with some few exceptions, is competent to delivery with safety; and in proof of this, among other illustrations, without referring to comparative physiology, reference need only be made to those unfortunate women, who, to avoid the shame of illegitimate pregnancy, conceal the birth of their children: they, unassisted and exposed to every casualty, rarely, as regards the act of parturition, do otherwise than well. No importunities, no desire of economising time, should prevail on the accoucheur to interfere with the progress of natural labour: he might, perhaps, should flooding occur through his officiousness, presume on his competency to restrain it; he would, however, have incurred an awful responsibility, and one that, as it implicates the safety of the patient, he is not justified in incurring. There is a prevailing, but a most mistaken opinion, that obstetric reputation is to be inferred from expedition: this may be probably influential with young aspirants to fame, and be most influential with those who are the least prepared to meet the untoward circumstances that such imprudence may provoke. I have known even experienced practitioners, while engaged in these anxious and arduous duties, to have their minds so absorbed with the desire of obtaining a speedy delivery, as the most effectual security against impending danger, that they have involved themselves in the dilemma they wished to avoid; for, delivery effected, they have had to contend with an unmanageable hemorrhage, which, by letting the uterus properly participate in the expulsion of its contents, might have been avoided.

The following case, in which hemorrhage had occurred in three successive labours, the progress of which were hastened, and did not in three subsequent deliveries, in which no officious interference was permitted, may be given as a practical illustration of the foregoing remarks.

Mrs. P., a lady of more mind than physical power, was, in consequence of her accustomed accoucheur retiring from ill health, committed to my care, with an anxious solicitude for her safety. In her three first accouchements, she had suffered, as reported, from hourglass contraction, accompanied each time with hemorrhage to an extent that endangered her life. I delivered her in three subsequent labours by the non-interference system, (the propriety of which I have been endeavouring to enforce,) with no other occurrence than what usually attends parturition.

I mention this case in illustration, as in it, by over anxiety or from some other cause, the previous labours had been hastened; and, hemorrhage having each time occurred, as I was informed, in her previous accouchements, I was prepared to expect a similar occurrence; for a kind of predisposition might have been thus established.

This paper is addressed to young practitioners chiefly, and the older will excuse me for repeating what is familiar to them: it is but a recognition of the axiom that the most efficient agent in preventing and restraining uterine hemorrhage is uterine contraction. This, I am aware, is urgently impressed on the minds of students by our public teachers; but good precepts are of little avail, if we permit a breach in the observance; and we all know that the mere admission of a truth is very different from the lively impressions which reiterated cases make upon the mind, and the effects which their remembrance produces in general practice.

